# Clinical expression of cystic fibrosis in a large cohort of Italian siblings

**DOI:** 10.1186/s12890-018-0766-6

**Published:** 2018-12-22

**Authors:** Vito Terlizzi, Marco Lucarelli, Donatello Salvatore, Adriano Angioni, Arianna Bisogno, Cesare Braggion, Roberto Buzzetti, Vincenzo Carnovale, Rosaria Casciaro, Giuseppe Castaldo, Natalia Cirilli, Mirella Collura, Carla Colombo, Antonella Miriam Di Lullo, Ausilia Elce, Vincenzina Lucidi, Elisa Madarena, Rita Padoan, Serena Quattrucci, Valeria Raia, Manuela Seia, Lisa Termini, Federica Zarrilli

**Affiliations:** 1 Dipartimento di Pediatria, Centro Regionale Toscano per la Fibrosi Cistica, Azienda Ospedaliero-Universitaria Meyer, Viale Gaetano Pieraccini 24, 50139 Florence, Italy; 2 Dipartimento di Biotecnologie Cellulari ed Ematologia, Istituto Pasteur Fondazione Cenci Bolognetti, Sapienza Università e Policlinico Umberto I, Rome, Italy; 3 Centro Regionale Fibrosi Cistica, Centro Pediatrico Bambino Gesù Basilicata, AOR San Carlo, Potenza, Italy; 4 Laboratorio di Genetica Medica, Ospedale Pediatrico Bambino Gesù, Rome, Italy; 5 Centro Regionale Fibrosi Cistica, Fondazione IRCCS Ca’ Granda, Ospedale Maggiore Policlinico, Università degli Studi di Milano, Milan, Italy; 6 Freelance Epidemiologist, Bergamo, Italy; 7 Centro Regionale Fibrosi Cistica Adulti, Dipartimento di Scienze Mediche Traslazionali, Università di Napoli Federico II, Naples, Italy; 8 Centro Regionale Fibrosi Cistica, U.O.C. Pneumologia, IRCCS G. Gaslini, Genua, Italy; 9 Dipartimento di Medicina Molecolare e Biotecnologie Mediche, Università di Napoli Federico II, Naples, Italy; 10 CEINGE-Biotecnologie avanzate, Naples, Italy; 11 Centro Regionale Fibrosi Cistica, Dipartimento Materno-Infantile, Ospedali Riuniti Ancona, Ancona, Italy; 12 CRR Fibrosi Cistica, Ospedale dei Bambini, ARNAS Civico, Palermo, Italy; 13 Dipartimento di Neuroscienze, Scienze Riproduttive ed Odontostomatologica, Università di Napoli Federico II, Naples, Italy; 14 Università Telematica Pegaso, Naples, Italy; 15 Unità Regionale di Fibrosi Cistica, IRCCS Ospedale Pediatrico Bambino Gesù, Rome, Italy; 16 Centro Regionale Fibrosi Cistica, Ospedale Giovanni Paolo II, Lamezia, Italy; 17 Centro Regionale di supporto Fibrosi Cistica, Dipartimento di Pediatria, Università di Brescia, AO Spedali Civili, Brescia, Italy; 18 Centro Fibrosi Cistica Regione Lazio, Dipartimento di Pediatria e Neuropsichiatria Infantile, Sapienza Università-Policlinico Umberto I, Rome, Italy; 19 Centro Regionale Fibrosi Cistica, Sezione Pediatrica, Dipartimento di Scienze Mediche Traslazionali, Università di Napoli Federico II, Naples, Italy; 20 Laboratorio di Genetica Medica, Fondazione IRCCS Policlinico Ca’ Granda Ospedale, Milan, Italy; 21 Ospedale dei Bambini G. Di Cristina, Centro Regionale Fibrosi Cistica, Palermo, Italy; 22 Dipartimento di Bioscienze e Territorio, Università del Molise, Isernia, Italy

**Keywords:** CFTR, Genotype, Phenotype, Modifier genes, FEV_1_, Pseudomonas aeruginosa

## Abstract

**Background:**

A clinical heterogeneity was reported in patients with Cystic Fibrosis (CF) with the same *CFTR* genotype and between siblings with CF.

**Methods:**

We investigated all clinical aspects in a cohort of 101 pairs of siblings with CF (including 6 triplets) followed since diagnosis.

**Results:**

Severe lung disease had a 22.2% concordance in sib-pairs, occurred early and the FEV_1_% at 12 years was predictive of the severity of lung disease in the adulthood. Similarly, CF liver disease occurred early (median: 15 years) and showed a concordance of 27.8% in sib-pairs suggesting a scarce contribution of genetic factors; in fact, only 2/15 patients with liver disease in discordant sib-pairs had a deficiency of alpha-1-antitrypsin (a known modifier gene of CF liver phenotype). CF related diabetes was found in 22 pairs (in 6 in both the siblings). It occurred later (median: 32.5 years) and is strongly associated with liver disease. Colonization by *P. aeruginosa* and nasal polyposis that required surgery had a concordance > 50% in sib-pairs and were poorly correlated to other clinical parameters. The pancreatic status was highly concordant in pairs of siblings (i.e., 95.1%) but a different pancreatic status was observed in patients with the same *CFTR* mutations. This suggests a close relationship of the pancreatic status with the “whole” *CFTR* genotype, including mutations in regulatory regions that may modulate the levels of *CFTR* expression. Finally, a severe course of CF was evident in a number of patients with pancreatic sufficiency.

**Conclusions:**

Physicians involved in care of patients with CF and in genetic counseling must be aware of the clinical heterogeneity of CF even in sib-pairs that, at the state of the art, is difficult to explain.

**Electronic supplementary material:**

The online version of this article (10.1186/s12890-018-0766-6) contains supplementary material, which is available to authorized users.

## Background

Cystic fibrosis (CF) is the most common, severe, autosomal recessive inherited disease among Caucasians [1]. It is usually characterized by elevated sweat chloride levels (SCL), pancreatic insufficiency (PI), progressive lung disease with chronic bacterial infections of lower airways and male infertility due to obstructive azoospermia. More than 2000 variants have been identified in the *cystic fibrosis transmembrane conductance regulator* (*CFTR*) gene so far (www.genet.sickkids.on.ca), but only few have been functionally characterized [2]. Some of them may be grouped in six classes according to the known effect on CFTR synthesis and/or function [2, 3]. However, although life-expectance and severity of the disease depend on the class of mutations [4, 5], there is a wide clinical heterogeneity in CF patients carrying the same *CFTR* genotype and even between siblings and twins with CF [6]. Several sources contribute to such variability as mutations in non-coding regions of the *CFTR* gene [7–9], intronic variants [9–11] and complex alleles [12, 13] making the genotype-phenotype relationship more complex.

In the last decade several studies explored the role of modifier genes of CF phenotype [14] for meconium ileus (MI) [15–17], CF-related diabetes (CFRD) [18], CF liver disease (CFLD) [19], lung disease [20, 21] and the colonization by *P. aeruginosa* [22]. However, environmental factors, such as the quality of health care, compliance to therapy [23], lifestyle, and socio-economic status [24] play a pivotal role in the outcome of CF.

The strong heterogeneity of CF impacts on the genetic counseling and on the reproductive planning of families that have CF affected children. In the present study, we evaluated a series of pairs of siblings with CF monitored since diagnosis to study the degree of clinical heterogeneity of the CF phenotype, assessing and correlating clinical aspects and complications of the disease.

## Methods

### Patients

The study population consisted of patients followed since diagnosis at 12 CF specialized care centers in Italy who met diagnostic criteria for CF [25] and had at least one sibling suffering from CF. According to the current Italian legislation, we obtained from all patients (or from their legal guardian) the informed consent to use anonymously clinical data for research purposes.

### Methods

Sweat chloride levels were tested according to guidelines [26]. We screened for a panel of mutations and for the most common *CFTR* rearrangements [27]; thus, we carried out gene sequencing (detection rate 95%) [28, 29] in patients in which one or both mutations resulted undetected after first level analysis [30]. We analyzed 7 intragenic *CFTR* short tandem repeats [31] to verify that both members of four sibling-pairs carrying only one known mutation had the same *CFTR* genotype. Molecular analysis revealed more than 50 different *CFTR* mutations in our patients and only for a half of them functional studies had defined the molecular effect and the class; furthermore, some of these latter mutations may combine more defects and can be attributed to more classes [2]. Thus, we did not perform correlations between the *CFTR* genotype and clinical parameters. However, Additional file 1: Table S1 provides the *CFTR* genotype of each patient included in the study.

Meconium ileus was defined using the criteria described [15]. The forced expiratory volume (FEV_1_), expressed as the percentage of predicted value for age, according to standardized reference equations for spirometry [32] was recorded. To avoid age-related differences between siblings of each pair, we recorded as current FEV_1_ the last value of the younger sibling and that of the older sibling at the same age. In the case of patients who had died or had undergone lung transplantation we recorded the last FEV_1_ and the value of the living or non-transplanted member of the sibling-pair at the same age. Given the inter-individual variability of FEV_1_ and the evolution of lung damage with age [33] the patients were classified as severe or mild according with the criteria by Schluchter et al., that take into account both the FEV_1_ value and age [34, 35]. The airway colonization by *P. aeruginosa* was identified by sputum or oropharyngeal swab culture. Chronic infection was defined according to the modified Leeds criteria [36].

Fecal pancreatic elastase was evaluated annually and at least 3 months before enrollment. Pancreatic sufficiency was defined on the basis of fecal pancreatic elastase-1 higher than 200 mcg/g measured in the absence of acute pancreatitis or gastrointestinal diseases. Pancreatitis was defined as acute or chronic according to the report from International study group of pediatric pancreatitis [37] excluding all known causes of pancreatitis. Liver disease was evaluated by means of clinical, biochemical or ultrasonography abnormalities recorded in two consecutive examinations within a 3-month period, in the absence of other causes of congenital or acquired chronic liver disease [38]. Patients were considered as affected by CFLD when they had liver cirrhosis, considered as the extreme phenotype to define liver disease in our study, with imaging techniques showing nodular hepatic parenchyma and signs of portal hypertension [17]. A glucose tolerance test was performed annually in all patients with CF and the diagnosis of CFRD was made according to the standard American Diabetes Association criteria [39]. Finally, the history of nasal polyposis requiring surgery was evaluated.

### Statistics

The concordance for disease (symptoms or complications) within sibling-pairs was calculated as the ratio between the number of pairs concordant for the symptom/complication and the number of pairs in which at least one member had the symptom/complication [15].

## Results and discussion

### Study population

We studied 208 patients with CF (median age: 30 years, range 12–61 years; 106 males); of these, 40 (19.2%) were diagnosed by newborn screening (NBS) and 168 (80.8%) by symptoms or by family history. All the 208 patients were over 12 years of age and 172/208 patients (82.7%) were over 18 years. The study included 95 pairs of siblings (22 pairs of females, 27 pairs of males and 46 pairs of one female and one male) and 6 sets of 3 siblings (2 including two males and one female, 2 including two females and one male and 2 including three females). In 80/101 pairs (79.2%) all the siblings were above 18 years of age.

### Pancreatic status

As shown in Table 1, in our pairs of siblings we found a concordance for PI of 95.1% (i.e., only in three sibling-pairs the pancreatic status resulted discordant) confirming the known correlation between the pancreatic status and the *CFTR* genotype. However, pairs of siblings with the same *CFTR* mutations may have PS or PI, and it is known that mutations like the R334W, the R347P, the 2789 + 5G > A [40], or the D1152H [41] may be associated with PS or PI. This means that the high degree of concordance for the pancreatic status found in siblings depends on the “whole” *CFTR* genotype (that is quite completely shared by siblings) including either mutations in the coding regions and variants in non-coding regions like the promoter or the intronic regions [7, 8] that together define the levels of residual activity of the CFTR protein at pancreatic level and thus may modulate the pancreatic status of each patient.

**Table 1:** Concordance within sib-pairs for PI and correlation between the pancreatic status and meconium ileus (MI), severity of lung disease, CF liver disease (CFLD), CF related diabetes (CFRD) and recurrent panreatitis (RP) in 101 pairs of siblings with CF. N: absence of the symptom/complication

Concordance within sib-pairs for PI (%)95.1				
		PI/PI	PI/PS	PS/PS
Number of sib-pairs		58	3	40
Meconium ileus:	MI/MI	4	0	0
	MI/N	5	0	2
	N/N	49	3	38
Lung disease:	Severe/severe	6	0	0
	Severe/mild	15	1	5
	Mild/mild	37	2	35
CF liver disease:	CFLD/CFLD	3	2	0
	CFLD/N	11	0	2
	N/N	44	1	38
CF related diabetes:	CFRD/CFRD	5	0	1
	CFRD/N	11	3	2
	N/N	42	0	37
Pancreatitis:	RP/RP	1	0	1
	RP/N	1	0	6
	N/N	56	3	33

Table 1 compares the clinical characteristics and the complications in sib-pairs with different pancreatic status. In sib-pairs with PI, MI, severe lung disease, CFLD and CFRD occurred more frequently (even if the small number of cases preclude a statistical comparison) according to the current literature [42, 43]. Recurrent pancreatitis was significantly more frequent in sib-pairs with PS in agreement with the concept that recurrent pancreatitis in patients with CF is due to ductal plugging that typically occurs in patients with PS [43, 44]. On the other hand, modifier genes other than *CFTR* may enhance the risk to develop recurrent/chronic pancreatitis [45, 46] explaining some cases of patients with PI that developed recurrent pancreatitis (Additional file 1: Table S1).

Interestingly, among the patients with PS we found MI in 2 cases, severe lung disease in 6 and complications such CFLD in 5 or CFRD in 7 cases (Additional file 1: Table S1). We are confident that our patients with PS would not develop PI later either because most patients with PS have at least one mild mutation and because their age is higher than that of patients with PI (mean 34.6 years, median 33 years for patients with PS versus a mean age of 28.0 years and a median of 25 years of patients with PI). Thus, a severe clinical phenotype may occur in a percentage of patients with PS. Considering that most patients with CF and PS are nowadays diagnosed by NBS, the presence of these complicated cases suggests that patients with PS would be monitored with the same care of patients with PI.

### Meconium ileus and DIOS

We found a 36.4% of concordance for MI in sib-pairs, i.e., 4 pairs were concordant for MI and 7 pairs were discordant suggesting that in addition to the *CFTR* genotype other non-genetic and genetic factors contribute to the development of MI. Among the genetic ones, in CFTR-deficient mice, that is an excellent model for human MI, three potential modifier loci for MI were found on chromosomes 1, 9 and 10, respectively [47]. Subsequent studies in humans identified at least 2 modifier loci for MI [15] and thus, the SLC4A4 gene was candidate as modifier genes for MI in patients with CF [48]. Furthermore, a role of KCNN4 as a modifier gene of MI was suggested [16, 17].

We found five patients with CF that had DIOS; such complication was revealed in both the members of two sib-pairs and in a member of a further pair (Additional file 1: Table S1). Interestingly, none of the five patients experienced MI, reinforcing the view that DIOS, once considered the adult equivalent of MI, has an independent etiology [15].

### Lung disease

**Fig. 1:**
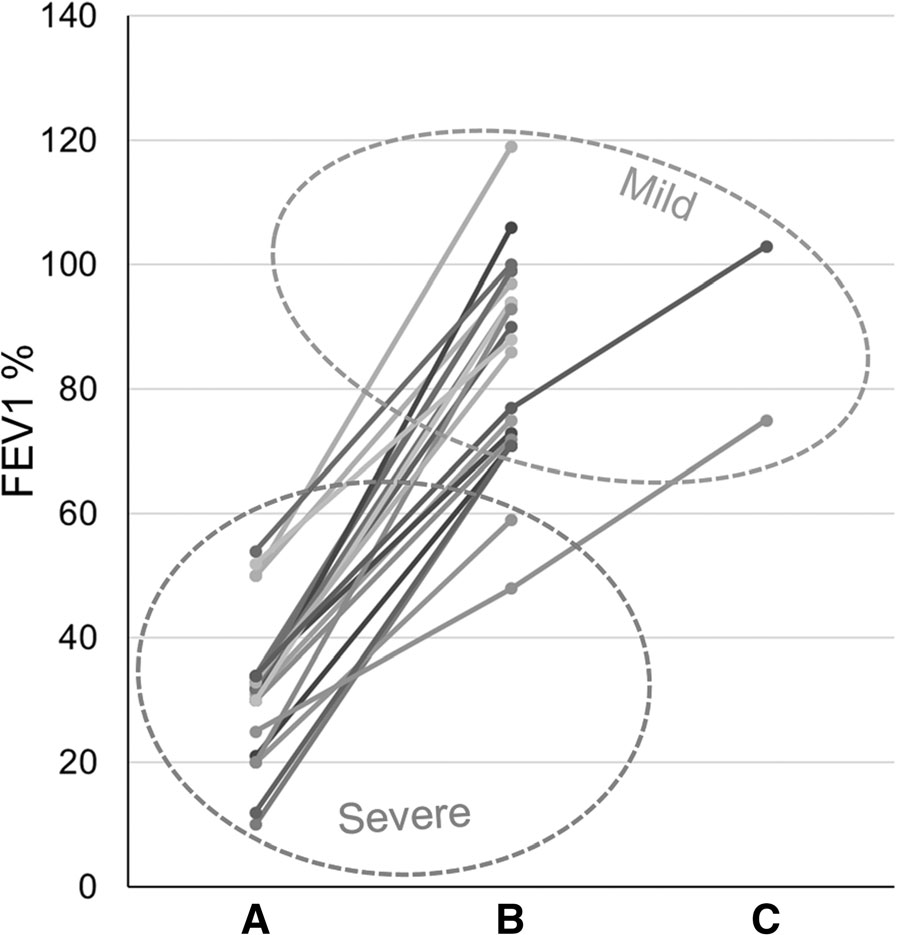
FEV_1_% of predicted for age in 21 pairs of siblings with cystic fibrosis discordant for the severity of lung disease. The classification as mild or severe was performed according to the criteria by Schluchter et al. [30] that take into account both the FEV_1_ value and age

We found 34/208 patients (16.4%) with severe lung disease. The concordance for severe lung disease was 22.2%, i.e., in 21 pairs the lung status was discordant (i.e., severe versus mild) between siblings (Fig. 1) while in 6 pairs both the siblings had a severe lung disease suggesting that environmental factors and genes inherited independently by *CFTR* contribute to the pathogenesis of severe lung disease. This agree with the results of Vanscoy et al. on 231 pairs of siblings [49], and of Collaco et al. [21], that concluded that genetic and environmental factors contribute equally to lung function in patients with CF. Finally, an excellent study on 6365 patients with CF revealed five loci that modulate the severity of lung disease in patients with CF [20]. The severe lung disease correlated with PI (e.g., all the 6 pairs of siblings with severe lung disease had PI, see Additional file 1: Table S1) and with *P. aeruginosa* colonization (see below).

**Fig. 2:**
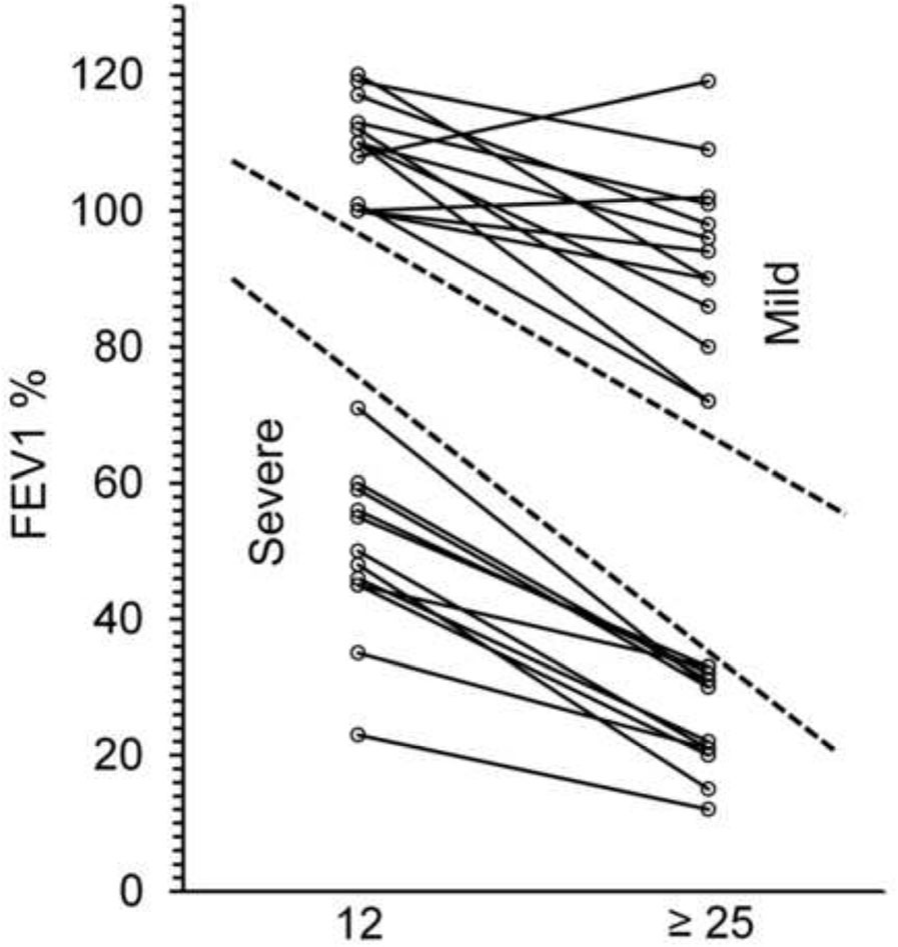
FEV_1_% of predicted for age in 25 patients with cystic fibrosis. For each patient we report the value measured at the age of 12 years and the current value at the age of 25 years or later. The classification of mild (13 cases) or severe (12 cases) was performed according to criteria by Schluchter et al. [30] that take into account both the FEV_1_ value and age

It is interesting to observe the longitudinal data of 25 patients from our study for which the values of FEV_1_ at the age of 12 years and at the age of 25 years or later were available (Fig. 2). Even if the number of cases is small, the classification of severe (*n* = 12) or mild (*n* = 13) lung disease performed at 12 years invariably coincides with that obtained in the adulthood in the same patient, suggesting that the FEV_1_% in young patients with CF is predictive of the lung function in the adult age.

On the other hand, it is known that structural lung damage is an early event in patients with CF [50]. This was revealed by high-resolution chest tomography [51] and by the presence of severe pulmonary inflammation and structural lung disease in still asymptomatic patients diagnosed by NBS [52].

### Colonization by Pseudomonas aeruginosa

We found colonization by *P. aeruginosa* in 92/208 (44.2%) patients with CF. Among our 101 pairs of siblings, we found colonization by *P. aeruginosa* in all the members of 34 sibling-pairs, among which the three siblings of two triplets and only one member of 20 sibling-pairs (and in two of three members of a triplet), with a high concordance for colonization (i.e., 61.8%). This result agrees with previous studies on 50 [52] and 11 [19] pairs of siblings. Interestingly, among our siblings discordant for colonization, in 11/21 cases both the siblings lived in the same house, and the sibling colonized by *P. aeruginosa* was colonized since at least 3 years. These data indicate a limited role of the environment in the colonization and confirms a contribution of *CFTR* genotype and genes inherited independently by *CFTR* that may predispose to colonization by *P. aeruginosa,* as reviewed by Cutting [3]. Nevertheless, we cannot rule out an early intervention (e.g. antibiotics) to prevent nosocomial infection in non-colonized siblings.

In patients with colonization there is a significantly higher occurrence of severe lung disease (data not shown). However, colonization is likely a consequence of the severe pulmonary damage more than a causal contribution. Indeed, the mean age of colonization in our patients was 18.5 years (median 22.6), while the occurrence of the structural lung damage is an early event in patients with CF (as discussed in the previous paragraph).

### CF liver disease

We found CFLD in 24/208 (11.5%) patients with CF. It is difficult to compare this figure with previous data because different parameters may be used to define CFLD (i.e., altered aminotransferase levels, focal biliary cirrhosis, US alterations, portal hypertension). As described in materials and methods, we used very stringent parameters to define CFLD and our results agree with the evidence that about 10% of patients with CF develop a severe liver damage and about 5% require liver transplantation. Our data confirm that CFLD is an early event since it was diagnosed at a mean age of 14.7 years (median 15 years old, range 7–27 years) in agreement with the view that CFLD peaks in adolescence [42].

CFLD was found in both the members of 5 sibling-pairs and in one member of 13 sibling-pairs (among which two triplets) and in two members of a further triplet. No previous data are available on the comparison of CFLD liver disease in siblings and in twins with CF, but the concordance for CFLD of 27.8% obtained in our sibling-pairs indicate a scarce contribution of genes in the pathogenesis of CFLD reinforcing the role of environmental, mostly still unknown, risk factors [38]. In fact, the largest two-stage control study (about 2000 patients with CF) on modifier genes of CFLD revealed mutations in the SERPIN1 gene encoding for alpha-1-antitrypsin only in about 2% of patients with CFLD [19]. In agreement, among the 15 sibling-pairs discordant for CFLD, only in 2 cases the sibling suffering from CFLD had levels of serum alpha-1-antitrypsin under the lower reference limit, compatible with the deficiency of the protein, while the siblings free from CFLD had normal levels of the protein.

### Cystic fibrosis related diabetes (CFRD)

We found CFRD in all the members of 6/101 sibling-pairs (among which one triplet) and in one member of 16/101 sibling-pairs. The concordance for CFRD was 27.3% and this figure compares with that of 18.0% reported in a population of 588 sibling-pairs [53]. These data, added to the concordance for CFRD of 73.0% obtained in 68 pairs of monozygotic twins with CF [53] that share 100% of DNA, support the view that genetic modifiers play a marginal role in the development of diabetes in patients with CF. This is also confirmed by the observation that the occurrence of CFRD is not an early complication in patients with CF [39]. In fact, the diagnosis of CFRD in our patients was performed at the mean age of 32.2 years and in more than a half of patients > 30 years. Furthermore, we excluded also the gender as a risk factor for CFRD: we found such complication in 13/106 males and in 15/102 females (*p* not significant). Most studies on larger populations reported the female gender as a risk factor for CFRD [42], but a more recent study on 588 sibling-pairs did not find a statistically significant role of gender as risk factor for CFRD [53].

As shown in Table 1, PI was significantly more frequent in sib-pairs with CFRD, confirming PI as a risk factor for CFRD since it causes a progressive pancreatic fibrosis that gradually damages the insulae [54]. However, observing that 7/28 patients with CFRD had PS (Additional file 1: Table S1), we suggest that also CF patients with PS must be included in the annual screening for CFRD based on the glucose tolerance test [42].

**Fig. 3:**
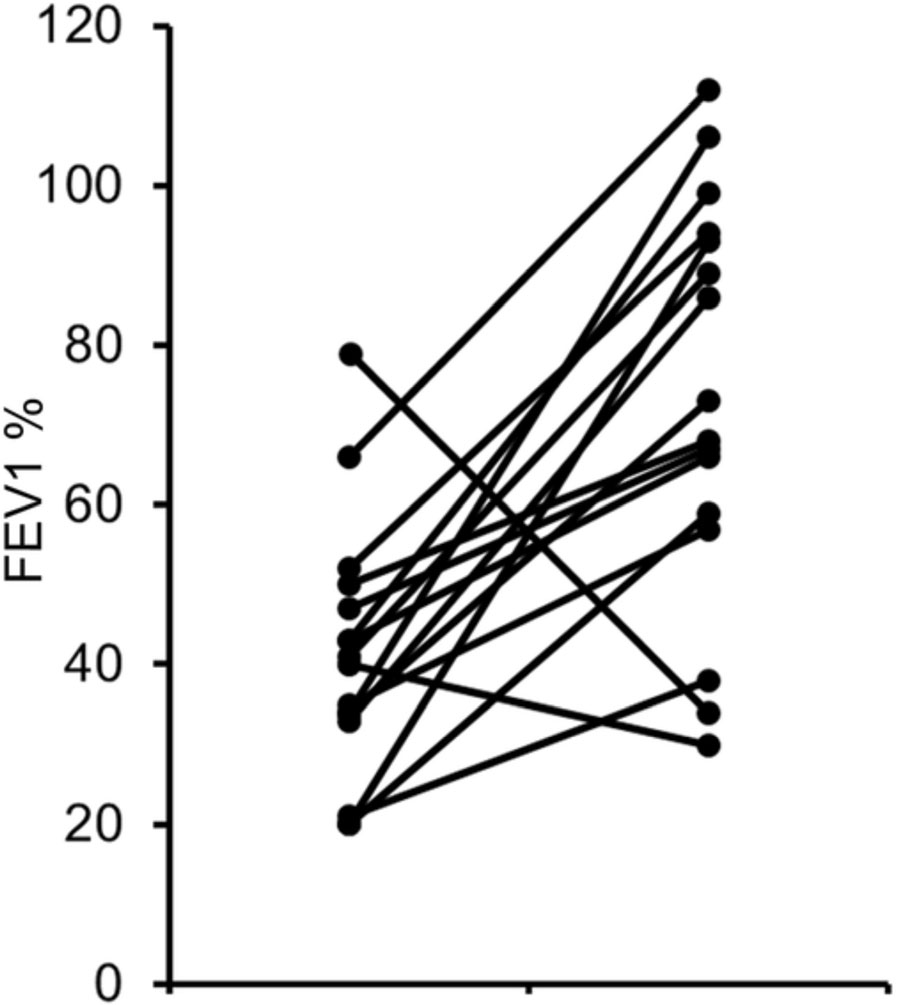
Current FEV_1_% of predicted for age in 15 pairs of siblings discordant for cystic fibrosis related diabetes (CFRD). **a** sibling with CFRD; **b** sibling free from CFRD. We reported as current FEV_1_ the last value of the younger sibling and that of the older sibling at the same age

In addition, CFLD contributes to the risk for CFRD [55] since we found a higher occurrence of CFLD among sib-pairs with CFRD than in those free from this complication. Finally, our data are in agreement with the view that severe lung disease is a risk factor for CFRD in turn [56, 57]. In fact, in 19 sib-pairs in which one or both patients had CFRD, in 12 cases one or both the siblings had a severe lung disease, a condition observed only in 15/79 pairs in which both the siblings were free from CFRD. This correlation is more evident in 16 sibling-pairs discordant for CFRD, in which the sibling with CFRD, at the same age, had the worst FEV_1_% (*p* = 0.004, Wilcoxon signed rank test) in 14/16 cases (Fig. 3).

### Nasal polyposis

We found nasal polyposis requiring surgery in both the siblings of 13 pairs and only in one sibling from 12 sibling-pairs (concordance for disease: 52.0%). There are no previous data on sibling pairs to which compare our results. The poor correlation of nasal polyposis to other clinical manifestations such as the pancreatic status and the severity of lung disease (data not shown) imply that modifier genes play a role in determining nasal polyposis. In fact, a recent study firstly described the potential role of interferon-related developmental regulator 1 (a known modifier gene for CF pulmonary disease severity) as a modifier gene of nasal polyposis in patients with CF [58].

## Conclusions

Our study confirms the clinical heterogeneity of CF also in a percentage of pairs of siblings with CF. Physicians involved in genetic counseling must be aware of a so wide and mostly unpredictable variability. Stochastic, environmental and genetic factors also independent by *CFTR* contribute to such variability, even if with a different weight on each variable (i.e., nasal polyposis and *P. aeruginosa* colonization may be more influenced by genetic factors while CFRD and CFLD or the severe lung disease may be influenced either by genetic and by environmental factors). However other variables like medical care (that significantly improved in the last years), strongly influenced the clinical expression of each patient of our cohort that includes CF patients in the wide range between 12 to 61 years. Some working hypotheses emerged from our study: i) the classification of the lung damage as severe or mild based on the FEV_1_% assessed at the age of 12 years coincides with that obtained in the adulthood; ii) CFRD is influenced by the severity of liver disease; iii) a severe course of the disease (including the occurrence of complications) may occur in a percentage of patients with CF and PS suggesting that also such cases, usually revealed by NBS and once classified as mild CF, should be monitored as the patients with PI.

## Additional file

Additional file 1: **Table S1.** Clinical and genetic data of the 208 patients with CF included in the study. (XLSX 44 kb)

## Abbreviations

CF: Cystic fibrosis
CFLD: CF liver disease.
CFRD: CF-related diabetes
CFTR: Cystic fibrosis transmembrane conductance regulator
MI: Meconium ileus
PI: Pancreatic insufficiency
PS: Pancreatic sufficiency
SCL: Sweat chloride levels
